# High‐dose sinomenine attenuates ischemia/reperfusion‐induced hepatic inflammation and oxidative stress in rats with diabetes mellitus

**DOI:** 10.1002/iid3.1271

**Published:** 2024-06-18

**Authors:** Bo Hui, Xiaogang Zhang, Dinghui Dong, Yantao Shu, Ren Li, Zhengan Yang

**Affiliations:** ^1^ Department of General Surgery Unit‐4 The Second Affiliated Hospital of Xi'an Jiaotong University Xi'an China; ^2^ Department of Hepatobiliary Surgery The First Affiliated Hospital of Xi'an Jiaotong University Xi'an China

**Keywords:** hepatic ischemia reperfusion, inflammation, oxidative stress, sinomenine

## Abstract

**Introduction:**

Ischemia‐reperfusion (I/R) injury, resulting from blood flow interruption and its subsequent restoration, is a prevalent complication in liver surgery. The liver, as a crucial organ for carbohydrate and lipid metabolism, exhibits decreased tolerance to hepatic I/R in patients with diabetes mellitus (DM), resulting in a significant increase in hepatic dysfunction following surgery. This may be attributed to elevated oxidative stress and inflammation. Our prior research established sinomenine's (SIN) protective role against hepatic I/R injury. Nevertheless, the impact of SIN on hepatic I/R injury in DM rats remains unexplored.

**Objective and Methods:**

This study aimed to investigate the therapeutic potential of SIN in hepatic I/R injury in DM rats and elucidate its mechanism. Diabetic and hepatic I/R injury models were established in rats through high‐fat/sugar diet, streptozotocin injection, and hepatic blood flow occlusion. Liver function, oxidative stress, inflammatory reaction, histopathology, and Nrf‐2/HO‐1 signaling pathway were evaluated by using UV spectrophotometry, biochemical assays, enzyme‐linked immunosorbent assay, hematoxylin‐eosin staining, and Western blot analysis.

**Results:**

High‐dose SIN (300 mg/kg) significantly attenuated hepatic I/R injury in DM rats, reducing serum activities of ALT and AST, decreasing the AST/ALT ratio, enhancing tissue contents of SOD and GSH‐Px, suppressing the levels of TNF‐α and IL‐6, improving the liver histopathology, and activating Nrf‐2/HO‐1 signaling by promoting Nrf‐2 trans‐location from cytoplasm to nucleus. Low‐dose SIN (100 mg/kg) was ineffective.

**Conclusions:**

This study demonstrates that high‐dose sinomenine's mitigates hepatic I/R‐induced inflammation and oxidative stress in diabetes mellitus (DM) rats via Nrf‐2/HO‐1 activation, suggesting its potential as a preventive strategy for hepatic I/R injury in DM patients.

## Graphical abstract

This study provides the first evidence that high‐dose sinomenine's (SIN) attenuates hepatic I/R‐induced inflammation and oxidative stress in diabetes mellitus (DM) rats, potentially through activation of the Nrf‐2/HO‐1 signaling pathway. Therefore, high‐dose SIN may represent an effective therapeutic strategy for DM patients suffering from hepatic I/R injury.

## 1 INTRODUCTION

Hepatic ischemia‐reperfusion (I/R), a major form of graft dysfunction, causes significant morbidity and mortality after the operation of liver transplantation (LTx). Ischemia is inevitable in the course of LTx, the subsequent reperfusion leads to severe hepatic inflammatory response, oxidative stress, as well as tissue injury.^1^, ^2^ Diabetes mellitus (DM), a prevalent metabolic condition, poses a profound impact on global health systems, resulting in significant consequences for individual health.^3^ Due to insulin resistance or absolute insufficiency of secretion, DM patients are mostly accompanied by severe glucose and lipid metabolism disorders and elevated stress levels, and about 20% of patients are accompanied by abnormal liver function.^4^, ^5^ The liver is a key organ for glycolipid metabolism, early research has proven both the physiological and structural abnormalities in diabetic liver.^6^ Some studies have reported that patients with DM have decreased tolerance to hepatic I/R injury, and the incidence of hepatic dysfunction after liver surgery is significantly increased. The mechanism may be related to high levels of oxidative stress and inflammation.^7^

Sinomenine (SIN) is an alkaloid isolated from traditional medical herb Sinomenium acutum, which exerts many pharmacological effects including antioxidative, anti‐inflammatory, immunosuppressive, analgesic, and antiapoptotic benefits.^8^, ^9^ SIN's protective role against inflammation and oxidation has been confirmed in female rats with gestational diabetes through the activation of TLR4/MyD88/NF‐κB signaling.^10^ SIN was shown to protect renal cells and decrease renal tissue damage in diabetic rats via inhibiting oxidative stress, apoptosis, as well as fibrosis.^11^ Also, SIN reduced diabetic nephropathy‐caused glomerular endothelial dysfunction.^12^ Additionally, SIN significantly improves cardiac function in diabetic rats by deactivating NF‐κB and blocking inflammatory factor‐induced immunoreactions.^13^ SIN alleviates diabetic peripheral neuropathic pain,^14^, ^15^ and confers cognitive protection by curtailing hippocampal neuron ferroptosis.^16^ Moreover, SIN has been found to play significant protective roles against I/R‐induced multiple organ injuries, including the liver,^17^ brain,^18^ heart,^19^ and kidney.^20^ However, whether SIN also exerts protective effects in diabetic rats with hepatic I/R injury has never been explored. Based on our previous results that SIN could attenuate I/R‐induced hepatic injury in healthy rats,^17^ this study aims to further detect if SIN could also serve a protective role in diabetes rats after hepatic I/R and what's the underlying mechanism. Considering diabetes is reaching epidemic levels among the adult population, clarificating this question would greatly reveal the preventive value of SIN in clinical practice.

## 2 METHODS AND MATERIALS

### 2.1 Animals

Adult male Sprague‐Dawley rats (8‐week‐old, 200 ~ 250 g, license number: SCXK [Shan] 2018‐001) were obtained from Experimental Animal Center of Xi'an Jiaotong University. They were maintained in controlled environments with a temperature of 25°C, a humidity of ~65%, and a cycle of 12‐h light/12‐h dark. Rats had standard chow and water freely. The use of rats and associated experimental procedures were validated by the Animal Care and Use Committee of the Second Affiliated Hospital of Xi'an Jiaotong University, and strict compliance was made with the Guide for the Care and Use of Laboratory Animals published by the National Research Council. This study was approved by Animal Ethics Committee of the Second Affiliated Hospital of Xi'an Jiaotong University.

### 2.2 Establishment of DM rat model

Rats received a diet consisting of high levels of sugar and fat (10% sucrose and 10% lard) for a period of 4 weeks. This was followed by an injection into their peritoneal cavity of 50 mg/kg of a 1% streptozotocin solution (Cat# 572201; Sigma‐Aldrich). Three days later, tail vein blood glucose was monitored, with a random blood sugar level of >16.7 mmol/L serving as a criterion for competent model establishment.

### 2.3 Grouping and SIN Pretreatment

Twenty DM rats were randomly classified as follows: I. the sham operation group (DM, *n* = 5); II. the hepatic I/R group (DM + I/R, *n* = 5); III. the hepatic I/R group pretreated with low‐dose SIN (DM + I/R + SIN/100, *n* = 5); IV. the hepatic I/R group pretreated with high‐dose SIN (DM + I/R + SIN/300, n = 5). SIN (Cat# HY‐15122A; MedChemExpress) was freshly prepared in 0.9% sterile saline containing 1% dimethylsulfoxide (DMSO) before injection. The prepared SIN at 100 mg/kg or 300 mg/kg was administered into rats via intraperitoneal (i.p.) injection once per day for five consecutive days before I/R induction. The dose of SIN was selected based on several previously published studies.^17^, ^21^ Rats in group I and II were injected with normal sterile saline at the same frequency. Rats received daily i.p. injections of SIN at 100 or 300 mg/kg for five consecutive days before I/R insult. Rats in groups I and II were given injections of sterile saline at the same frequency for comparison.

### 2.4 Induction of hepatic I/R

Anesthesia was administered to rats via i.p. injection of thiopental at a dose of 0.05 g/kg b.w., and then midline laparotomy was conducted. Hepatic I/R was induced based on a previous research.^17^ In brief, hepatic ischemia was created by using a microvascular clip and lasted for 1 h, as confirmed by the pale appearance of the clamped lobes. The clip was then discreetly displaced to permit a 3‐h blood reperfusion. Anesthesia was administered to rats before blood sample collection. Serum isolation was achieved by centrifugation. Following euthanasia by carbon dioxide asphyxiation, liver tissues were gathered. All surgical procedures were conducted under strictly sterile conditions.

### 2.5 Measurement of liver function

The serum activity of aspartate aminotransferase (AST) was measured with AST Assay Kit (Cat# C010‐3‐1; Jiancheng Bioengineering Institute). The AST in the serum (10 μL) catalyzed the amino transfer reaction between l‐aspartate and α‐ketoglutarate, producing oxaloacetate and l‐glutamate. In the presence of NADH and malate dehydrogenase (MDH), oxaloacetate was reduced to l‐malate, and NADH was oxidized to NAD^+^, resulting in a decrease in the optical absorption value at 340 nm. By monitoring the rate of decrease in optical absorption at 340 nm, the activity of AST was determined.

The serum activity of alanine aminotransferase (ALT) was measured with ALT Assay Kit (Cat# C009‐3‐1; Jiancheng Bioengineering Institute). The ALT in the serum (10 μL) catalyzed the amino transfer reaction between l‐alanine and α‐ketoglutarate, resulting in the formation of pyruvate and glutamate. Under the catalysis of NADH and lactate dehydrogenase (LDH), pyruvate reacted to produce lactate and NAD^+^. NADH had a specific absorption peak at 340 nm, and the rate of its oxidation was proportional to the activity of ALT in the serum. By measuring the rate of decrease in NADH absorbance at 340 nm, the activity of ALT was calculated.

### 2.6 Measurement of superoxide dismutase (SOD) and glutathione peroxidase (GSH‐Px)

SOD and GSH‐Px levels in the hepatic tissues were measured with SOD Assay Kit (WST‐1 method, Cat# A001‐3‐2; Jiancheng Bioengineering Institute) and GSH‐Px Assay Kit (Colorimetric method, Cat# A005‐1‐2; Jiancheng Bioengineering Institute), respectively, according to the manufacturer's instruction. Briefly, accurately weigh the hepatic tissue, and add 9 times the volume of physiological saline according to the ratio of weight (g) to volume (ml) of 1:9. Then, cut the tissue into small pieces and prepare a homogenate in an ice‐water bath. Centrifuge at 3000 rpm for 15 min, and collect the supernatant, which is the 10% homogenate supernatant ready for testing. For SOD, mix 20 μL of the supernatant with the SOD reagent kit, incubate at 37°C for 20 min, and read the absorbance at 450 nm using a microplate reader (Model 550; Bio‐Rad Laboratories, Inc.). For GSH‐Px, mix 0.2 mL of the supernatant with the GSH‐PX reagent kit, centrifuge at 4000 rpm for 10 min, and collect 1 mL supernatant, which was then mixed well with the kit, incubated at room temperature for 15 min, and read the absorbance at 412 nm.

### 2.7 ELISA

TNF‐α and IL‐6 expressions in hepatic tissues were measured with TNF‐α ELISA Kit (Cat# F16960; Westang Biotechnology Ltd) and IL‐6 ELISA Kit (Cat# F15870; Westang Biotechnology Ltd.), respectively, according to the manufacturer's instruction. Briefly, add 100 μL of standard or test sample to each well. Mix the reaction plate thoroughly and incubate at 37°C for 40 min. Wash the reaction plate thoroughly with washing solution 4–6 times and blot dry on filter paper. Add 50 μL of distilled water and the first antibody working solution to each well (excluding the blank). Mix the reaction plate thoroughly and incubate at 37°C for 20 min. Wash the reaction plate again. Add 100 μL of enzyme‐labeled antibody working solution to each well. Place the reaction plate at 37°C for 10 min. Wash the reaction plate again. Add 100 μL of substrate working solution to each well and incubate in the dark at 37°C for 15 min. Add 100 μL of stop solution to each well and mix well. Measure the absorbance at 450 nm using a microplate reader (Model 550; Bio‐Rad Laboratories, Inc.) within 30 min.

### 2.8 Hematoxylin‐eosin histological staining

Rat liver tissues were excised and promptly immersed in 10% formalin fixative. Subsequently, dehydrate the tissue by using graded concentrations of ethanol solutions (70%, 80%, 95%, and 100%). Clear the dehydrated tissue in xylene for 20 min, followed by a further immersion in fresh xylene for 20 min. Immerse the cleared tissue in molten paraffin and embed it within a paraffin block. Utilize a microtome to slice the embedded tissue block into 5‐μm thickness sections. De‐wax the sections in xylene and then hydrate them through graded concentrations of ethanol solutions. Stain the sections in hematoxylin, differentiate them with acidified ethanol, and subsequently stain with eosin. Repeat the dehydration and clearing process using ethanol and xylene. Allow the sections to air‐dry in a ventilated area before sealing them with neutral balsam. The histopathological changes were observed under a light microscope (Olympus IX51; Olympus Corporation).

### 2.9 Western blot analysis

Total proteins were extracted from the liver tissues by using with RIPA lysis buffer (50 mM Tris‐HCl pH 7.4, 150 mM NaCl, 1% NP‐40, 0.1% SDS). Protein concentrations were measured with Enhanced BCA Protein Assay Kit (Cat# P0010; Beyotime Biotechnology). Each sample containing 30 μg proteins was loaded onto 7.5%–10% SDS‐PAGE gels, then relocated onto polyvinylidene fluoride (PVDF) membranes (Millipore). Membranes were incubated with 5% fat‐free milk at room temperature for 1 h to block nonspecific bindings, followed by incubation with corresponding primary antibodies (Table 1) overnight at 4°C with rotation. The following day, membranes were washed with Tris‐buffered saline containing 0.05% Tween 20 three times, then incubated with HRP‐coupled secondary antibodies (Table 2) at room temperature for 1 h. Ultimately, an enhanced chemiluminescence kit (Millipore) was used to develop membrane using Image Quant LAS 4000 (GE Healthcare). GAPDH functioned as an internal control for protein loading.

**Table 1:** Summarization of the primary antibodies used in this study.

Target genes	Catalog no	Host	Vendor	Dilution for WB
Nrf‐2	33649	Rabbit	Cell signaling technology	1:1000
HO‐1	43966	Rabbit	Cell signaling technology	1:1000
GAPDH	sc‐365062	Mouse	Santa cruz biotechnology	1:200

**Table 2:** Summarization of the secondary antibodies used in this study.

Secondary ab	Catalog no	Vendor	Dilution for WB
Bovine anti‐rabbit IgG‐HRP	sc‐2370	Santa cruz biotechnology	1:500
Bovine anti‐mouse IgG‐HRP	sc‐2371	Santa cruz biotechnology	1:500

### 2.10 Extraction of cytoplasmic and nuclear protein

The cytoplasmic protein and nuclear protein were separated with Nuclear and Cytoplasmic Protein Extraction Kit (Cat# P0027; Beyotime Biotechnology) according to the manufacturer's instruction. Briefly, the rat liver tissues were cut into as fine fragments as possible. Mix an appropriate amount of cytoplasmic protein extraction reagent A (200 μL) and B (10 μL) in a ratio of 20:1, and add PMSF to a final concentration of 1 mM to prepare the tissue homogenate. Mix the tissue and the homogenate in a ratio of 200 μL of homogenate per 60 mg of tissue and homogenize thoroughly in a glass homogenizer on ice. Transfer the homogenate to a plastic centrifuge tube and incubate on ice for 15 min. Centrifuge at 4°C and 1500 g for 5 min. Transfer the supernatant to a pre‐cooled plastic tube, which is the partially extracted cytoplasmic protein. For the cell pellet, add 200 μL of cytoplasmic protein extraction reagent A containing PMSF to every 20 μL of cell pellet. Vortex at the highest speed for 5 s to completely suspend and disperse the cell pellet. Incubate on ice for 10 min. Add 10 μL of cytoplasmic protein extraction reagent B. Vortex at the highest speed for 5 s, incubate on ice for 1 min. Vortex at the highest speed for 5 s, and centrifuge at 4°C and 15000 g for 5 min. Immediately aspirate the supernatant into a pre‐cooled plastic tube, which is the partially extracted cytoplasmic protein. For the pellet, aspirate the residual supernatant completely and add 50 μL of nuclear protein extraction reagent containing PMSF. Vortex at the highest speed for 30 s to completely suspend and disperse the cell pellet. Return to ice bath and vortex at high speed for 30 s every 2 min for a total of 30 min. Centrifuge at 4°C and 15000 g for 10 min. Immediately aspirate the supernatant into a pre‐cooled plastic tube, which is the extracted nuclear protein. The cytoplasmic proteins obtained from several extraction steps can be combined. The variation of Nrf‐2 expression was detected by Western blot analysis.

### 2.11 Statistical analysis

Data analysis was carried out with SPSS Statistics (version 20.0; Statistical Package for the Social Sciences Inc.). One‐way analysis of variance (ANOVA) was employed for multiple comparisons, and SNK‐*q* was applied for pairwise comparisons. Data are presented as the means ± standard deviation. A *p*‐value less than .05 demonstrates a significant difference statistically.

## 3 RESULTS

### 3.1 High‐Dose SIN improves the liver function in DM rats

**Figure 1:**
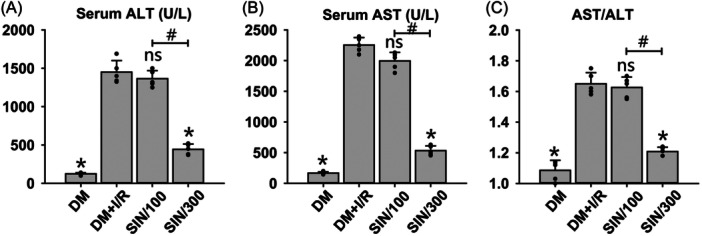
Evaluation of the hepatic function after I/R with/without SIN pretreatment in DM rats. The serum ALT (A) and AST (B) activities were measured by biochemical assays. (C) The ratio of AST to ALT was calculated. ANOVA was employed for multiple comparisons, followed by SNK‐*q* for pairwise comparisons. Bars represent means ± SD, *n* = 5 independent rats. *p < .05 versus DM + I/R group; ^#^ *p* < .05 versus DM + I/R + SIN/100 group; *ns*, not significant.

We initially evaluated liver function by assessing the activity of transaminases, specifically ALT and AST, in the serum of DM rats. Results showed that I/R significantly elevated the serum contents of ALT (Figure 1A) and AST (Figure 1B), as well as the ratio of AST to ALT (Figure 1C) in DM rats (*p* < .05). High‐dose SIN significantly reversed the I/R‐caused upregulation of ALT, AST, and AST/ALT (*p* < .05). Compared to the DM + I/R group, low‐dose SIN did not cause significant changes in serum ALT and AST levels, as well as AST/ALT ratio. The initial findings indicate that high‐dose SIN may have a protective effect against hepatic injury caused by I/R in DM.

### 3.2 High‐dose SIN alleviates hepatic oxidative stress in DM rats

**Figure 2:**
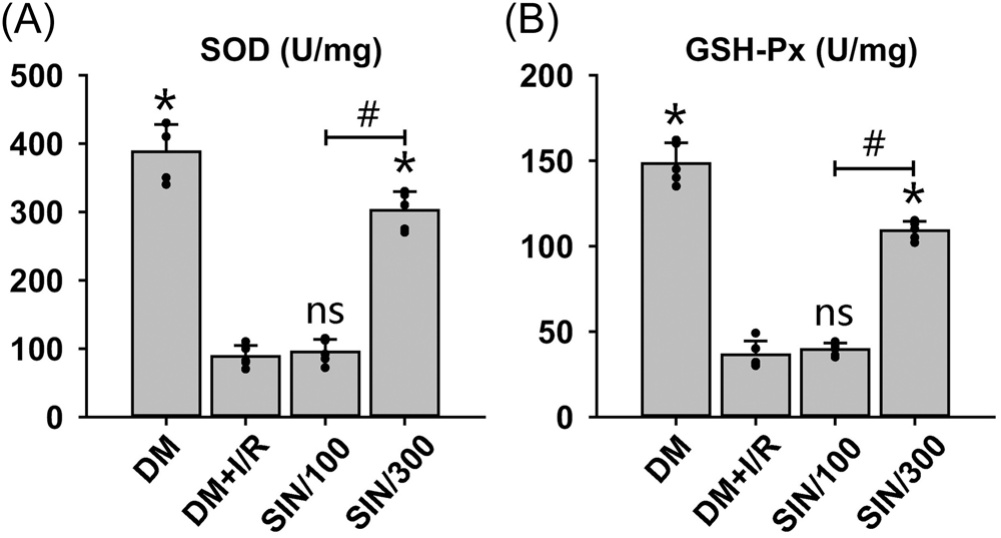
Evaluation of the oxidative stress after hepatic I/R with/without SIN pretreatment in DM rats. The SOD (A) and GSH‐Px (B) contents in liver tissues were measured by using biochemical assays. ANOVA was employed for multiple comparisons, followed by SNK‐*q* for pairwise comparisons. Bars show means ± SD, *n* = 5 independent rats. *p < .05 versus DM + I/R group; ^#^ *p* < .05 versus DM + I/R + SIN/100 group; *ns*, not significant.

We subsequently assessed the impact of SIN on I/R‐caused oxidative stress in diabetic rats. Data showed that I/R significantly reduced activities of SOD (Figure 2A) and GSH‐Px (Figure 2B) in DM rats (*p* < .05). High‐dose SIN significantly reversed I/R‐induced decrease of SOD and GSH‐Px activities (*p* < .05). Compared to the DM + I/R group, low‐dose SIN did not cause any changes in SOD and GSH‐Px activities. Collectively, the data indicate that high‐dose SIN exerts an antioxidative influence on hepatic injury caused by I/R in diabetic rats.

### 3.3 High‐dose SIN mitigates hepatic inflammation in DM rats

**Figure 3:**
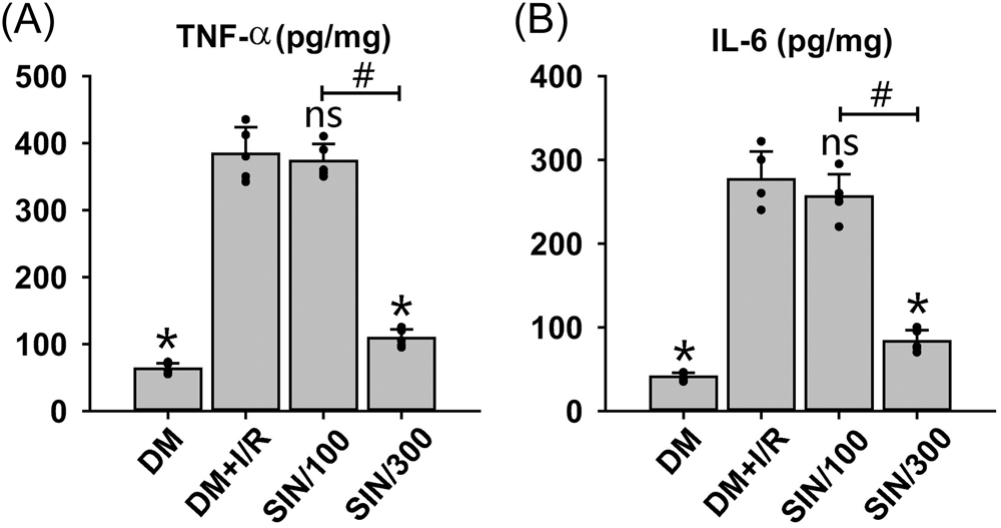
Evaluation of the inflammatory response after hepatic I/R with/without SIN pretreatment in DM rats. The levels of pro‐inflammatory cytokines TNF‐α (A) and IL‐6 (B) in liver tissues were measured by ELISA. ANOVA was employed for multiple comparisons, followed by SNK‐*q* for pairwise comparisons. Bars represent means ± SD, *n* = 5 independent rats. *p < .05 versus the sham operation group. *p < .05 versus DM + I/R group; ^#^ *p* < .05 versus DM + I/R + SIN/100 group; *ns*, not significant.

Next, we explored the impact of SIN on I/R‐caused inflammation in DM rats. Results showed that I/R significantly upregulated the tissue content of TNF‐α (Figure 3A) and IL‐6 (Figure 3B) in DM rats (*p* < .05). High‐dose SIN significantly reversed I/R‐induced elevation of TNF‐α and IL‐6 levels (*p* < .05), while low‐dose SIN did not alter the TNF‐α and IL‐6 levels compared to the DM + I/R group. Taken together, the data indicate that treatment with high‐dose SIN inhibited the I/R‐induced generation of pro‐inflammatory cytokines and alleviated the inflammatory response in diabetic rats.

### 3.4 High‐dose SIN reduces the severity of hepatic I/R injury in DM rats

**Figure 4:**
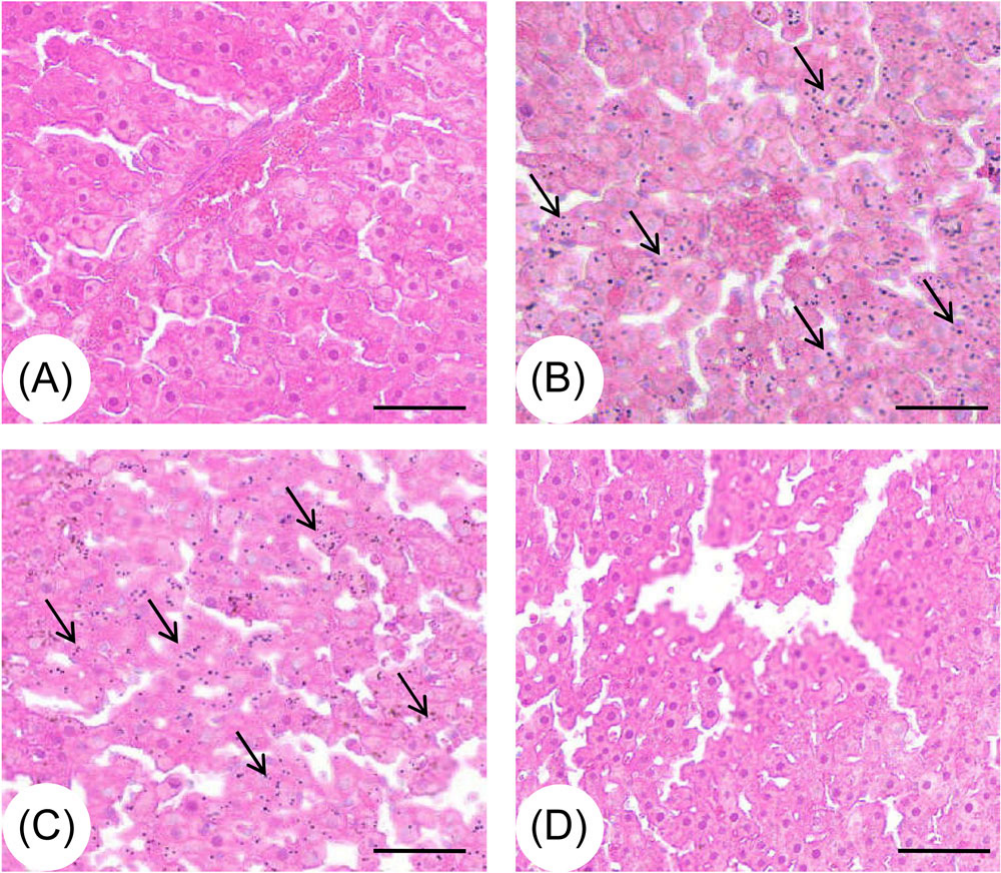
Evaluation of the pathological changes after hepatic I/R with/without SIN pretreatment in DM rats. The morphological changes of liver tissues were assessed by hematoxylin‐eosin staining. (A) Sham operation group. (B) I/R group. (C) I/R + low‐dose SIN group. (D) I/R + high‐dose SIN group. Black arrows point to inflammatory cell infiltration. Scar bars = 100 μm. Images are captured under 100 x magnification.

HE staining was performed to examine the pathological changes in DM rat liver. The data indicated that the hepatic sections in the DM group exhibited relatively intact hepatic lobules, lacking obvious inflammatory cell infiltration, disorganized fragments, vacuolation, or extensive necrosis (Figure 4A). The three I/R groups exhibited distinct histopathological alterations (Figure 4B–D). I/R caused significant damage to hepatic morphology, manifesting as significantly increased infiltration of inflammatory cells, hepatic edema, vacuole‐like changes, and hepatocellular necrosis (Figure 4B). In high‐dose SIN treatment group, the infiltration of inflammatory cells was remarkably reduced, the proportion of cytoplasmic vacuoles and necrotic cells was significantly decreased (Figure 4D). However, in low‐dose SIN treatment group, no significant improvement was observed when compared to DM + I/R group (Figure 4C). Collectively, these data demonstrate that high‐dose SIN could effectively attenuate I/R‐induced hepatic pathological changes in DM rats.

### 3.5 High‐dose SIN activates Nrf‐2/HO‐1 signaling pathway

**Figure 5:**
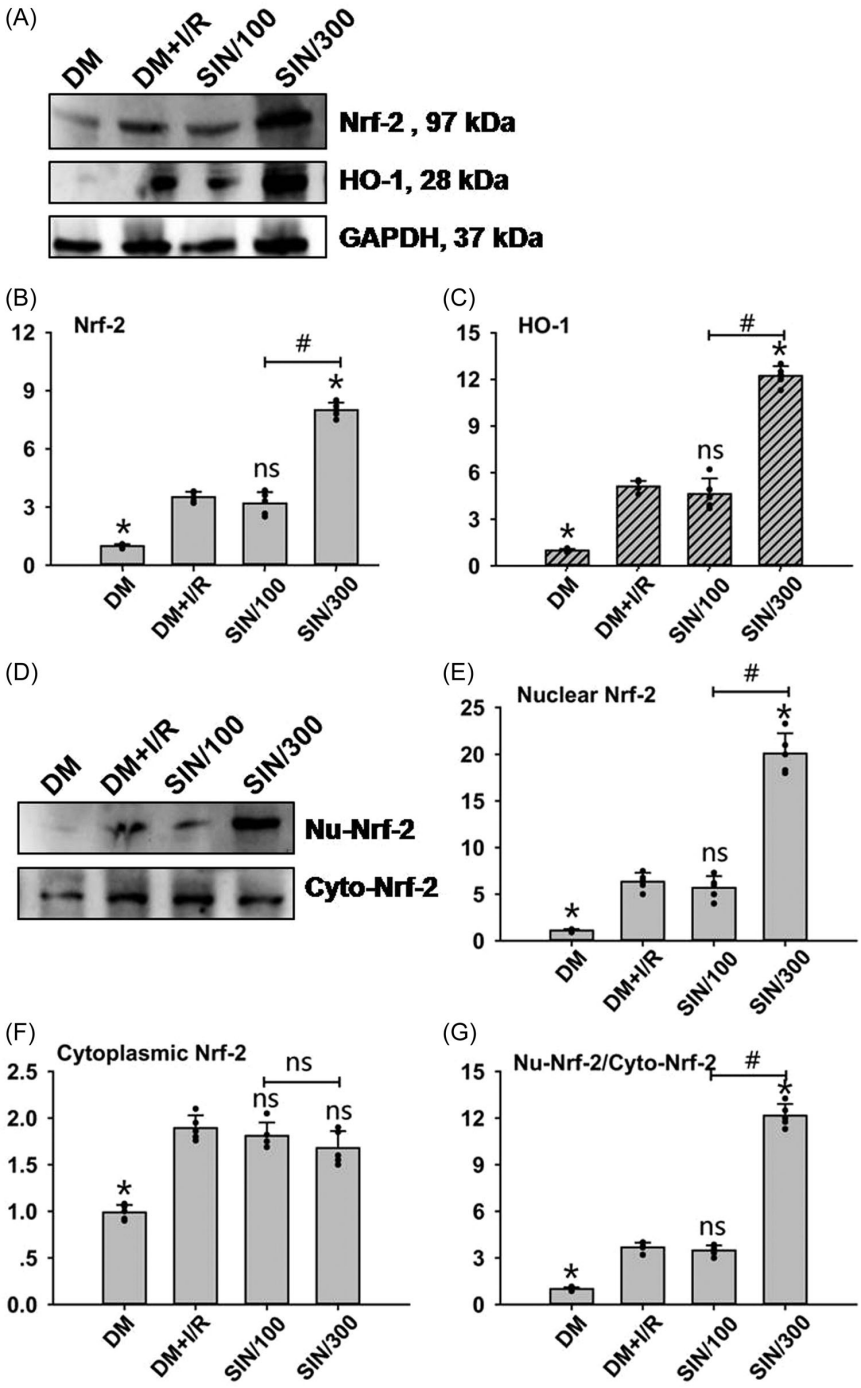
Evaluation of the Nrf‐2/HO‐1 pathway after hepatic I/R with/without SIN pretreatment in DM rats. The protein levels of total Nrf‐2, HO‐1, nuclear Nrf‐2 (Nu‐Nrf‐2), and cytoplasmic Nrf‐2 (Cyto‐Nrf‐2) in liver tissues were assessed by Western blot analysis. (A) Representative blots of Nrf‐2 and HO‐1 protein expression in liver tissues. Histograms showed the statistical results of Nrf‐2 (B) and HO‐1 (C). (D) Representative blots of Nu‐Nrf‐2 and Cyto‐Nrf‐2 protein expression. Histograms showed the statistical results of Nu‐Nrf‐2 (E) and Cyto‐Nrf‐2 (F). The ratio of Nu‐Nrf‐2 to Cyto‐Nrf‐2 was also calculated in (G). ANOVA was employed for multiple comparisons, followed by SNK‐*q* for pairwise comparisons. Bars represent means ± SD, *n* = 5 independent rats. *p < .05 versus DM + I/R group; ^#^ *p* < .05 versus DM + I/R + SIN/100 group; *ns*, not significant.

Lastly, we examined the expression of crucial proteins within the Nrf‐2/HO‐1 signaling pathway to elucidate the mechanisms underlying SIN's ability to mitigate oxidative stress and inflammation in hepatic I/R‐injured DM rats. Results showed that I/R itself could significantly up‐regulate the tissue protein levels of total Nrf‐2 and HO‐1 in DM rats (*p* < .05) (Figure 5A–C). High‐dose SIN further enhanced the I/R‐induced increase of total Nrf‐2 and HO‐1 protein levels (*p* < .05) (Figure 5A–C), while low‐dose SIN did not alter their expression when compared to those in the DM + I/R group (Figure 5A–C). The relocation of Nrf‐2 from cytoplasm to the nucleus is essential for the activation of Nrf‐2/HO‐1 signaling. Results showed that I/R alone significantly upregulated both the nuclear (Nu‐) and cytoplasmic (Cyto‐) Nrf‐2 expression, as well as the ratio of Nu‐/Cyto‐Nrf‐2 (*p* < .05) (Figure 5D–G). High‐dose SIN further enhanced Nu‐Nrf‐2 protein expression and the ratio of Nu‐/Cyto‐Nrf‐2 (*p* < .05) (Figure 5D–E,G), while showed no influence in Cyto‐Nrf‐2 protein expression compared to the DM + I/R group (Figure 5F). Low‐dose SIN did not significantly affect above mentioned changes compared to the DM + I/R group (Figure 5D–G). These data suggest that high‐dose SIN can further activate Nrf‐2/HO‐1 signaling by promoting the translocation of Nrf‐2 from cytoplasm to the nucleus in hepatic I/R‐injured DM rats.

## 4 DISCUSSION

The liver plays a pivotal role in oxidative and detoxifying processes, as well as free radical reactions. During various diseases, biomarkers of oxidative stress significantly increase in the liver, such as DM.^22^ I/R‐induced liver injury is a significant factor leading to morbidity and mortality in patients receiving liver surgery and transplantation.^23^ During hepatic ischemia, the absence of adequate blood supply and oxygen causes severe liver damage. Paradoxically, during the reperfusion phase, the production of harmful reactive oxygen species (ROS) and inflammatory mediators is enhanced. In diabetic patients, this damage is exacerbated.^24^ I/R injury stimulates Kupffer cells to produce ROS, pro‐inflammatory cytokines, and chemokines. This triggers an inflammatory reaction featured by neutrophil activation and promotes various pathological status including leukocyte infiltration, oxidative stress, inflammation, platelet activation, nitric oxide release, and apoptosis.^25^, ^26^

Elevated glucose levels significantly trigger nonenzymatic glycosylation and auto‐oxidative reactions, activate protein kinase‐C, and modulate the levels of inflammatory mediators.^27^ Previous research have established that high glucose‐induced oxidative stress contributes to diabetic inflammation.^28^ DM results in oxidative stress, and enhances inflammatory reaction caused by I/R injury. Cross‐sectional and longitudinal studies have linked diabetes with decreased liver function.^29^ Moreover, ROS‐induced oxidative stress exerted key roles in the development of diabetic complications.^30^ The present study demonstrates that pretreatment with high‐dose SIN alleviates hepatic I/R injury by inhibiting inflammation and oxidative stress, improving liver function and pathology in DM rats.

SIN is a pure alkaloid extracted from Sinomenium acutum, a Chinese medicinal plant. In recent years, numerous studies have investigated the benefits of SIN, which have been reported to include potent antioxidant, antiapoptotic, anti‐inflammatory, and immune regulatory activities.^31^, ^32^ SIN has been found to exert protective roles against I/R injury in multiple organs. For instance, SIN has been shown to protect the brain against cerebral I/R injury in ischemic rats via the anti‐inflammatory effect, which relieves acidosis, enhances energy metabolism, and reduces ASIC1a expression.^18^ SIN also exhibits an anti‐arrhythmia effect in I/R‐injured rats by altering oxidative stress and inflammatory reactions.^19^ A mechanistic study showed that SIN inhibits NF‐κB transcriptional activity to quell the kidney's I/R‐induced inflammatory response, while it mitigates MAPK signaling to forestall tubular cell apoptosis following I/R induction.^20^ Sinomenine confers protection against hepatic^17^ and myocardial I/R injury by preventing oxidative Stress, cellular apoptosis, and inflammation.^33^

Furthermore, SIN has been demonstrated to exhibit protective actions against various diabetic sequelae. For instance, SIN was observed to hinder the progression of diabetic nephropathy by influencing multiple genes and metabolites related pathways through network pharmacology and molecular docking analyses.^34^ SIN also exerts neuroprotective effects by curtailing hippocampal neuron ferroptosis and could be a potential candidate for preventing diabetic cognitive dysfunction.^16^ Additionally, SIN suppresses microglial activation and the release of inflammatory mediators, easing the pain associated with diabetic peripheral neuropathy.^14^

ALT, AST, and the ratio of AST/ALT are sensitive detection indicators of liver dysfunction, and commonly used in many clinical trials. TNF‐α and IL‐6 are critical pro‐inflammatory factors secreted by damaged hepatocytes and activated Kupffer cells. They play a significant role in the inflammatory response during hepatic I/R injury. Nrf‐2 is a key transcription factor and sustained in the cytoplasm via the interaction with Keap1. Upon stimulation, it trans‐locates into the nucleus to trigger transcription of antioxidant response element genes and regulate the expression of downstream target genes, including HO‐1, and other phases II antioxidant enzymes: SOD, GSH‐Px, and so forth.^35^ The Nrf‐2 antioxidant response pathway acts as the primary cellular defense mechanism against the cytotoxic effects of oxidative stress, and exerts protective actions against a range of hepatic disorders, including cholestatic liver injury, viral hepatitis, nonalcoholic fatty liver disease, and drug‐induced hepatic injury.^36^

The present study summarizes three key findings: hepatic I/R in DM rats leads to oxidative stress and inflammation due to decreased antioxidant enzymes and elevated pro‐inflammatory cytokines; pretreatment with high‐dose SIN mitigates hepatic I/R damage in diabetic rats by reducing oxidative stress, inflammation, and improving liver function and pathology; and high‐dose SIN activates Nrf‐2/HO‐1‐mediated endogenous antioxidant stress/anti‐inflammation mechanism in these rats. To the best of our knowledge, the current study represents the first to offer direct evidence demonstrating the protective effects of low‐dose SIN at 100 mg/kg b.w. are eliminated in diabetic rats, which has been previously proven effective in normal I/R rats.^17^ However, an increased dose of SIN at 300 mg/kg b.w. reverses the adverse effects of hepatic I/R and exhibits more protective effects within diabetic rats.

We should mention some limitations in our experimental study. Firstly, DM rats were terminated 3 h subsequent to reperfusion in the present study. We strongly recommend extending the observations of the recipients of SIN. Secondly, further investigation is required to study preventive effects of SIN pretreatment on liver function, including metabolic function, detoxification, and immune function. Thirdly, the lowest effective dose of SIN should be further clarified to minimize side effects. Finally, it is better to include an additional control group (nondiabetic group) to define the level of inflammation/oxidation stress specifically related to DM, which would require more animal samples. More research is desired to evaluate the clinical application of SIN in DM patients with I/R injury. After all, this is an exploratory experimental study that potentially carries all biases related to differences in metabolisms between rats and humans.

In conclusion, the present study demonstrates that high‐dose SIN exerts potent protective effects against hepatic I/R injury in DM rats. The anti‐inflammation and antioxidative properties of SIN, which are ascribed to activating Nrf‐2/HO‐1 signling, contribute significantly to its beneficial effects. These involved chemicals or molecules might be potential novel preventive agents or targets following liver transplantation in patients with DM.

## AUTHOR CONTRIBUTIONS

*Conceived and designed the experiments*: Bo Hui and Zhengan Yang. *Performed the experiments*: Bo Hui, Xiaogang Zhang, and Dinghui Dong. *Analyzed the data*: Bo Hui, Yantao Shu, and Ren Li. *Contributed reagents/materials/analysis tools*: Xiaogang Zhang, Dinghui Dong, Yantao Shu, and Ren Li. *Wrote and revised the paper*: Bo Hui and Zhengan Yang.

## CONFLICT OF INTEREST STATEMENT

The authors declare no conflict of interest.

## Data Availability

The authors have nothing to report.
